# Effect of innovation capacity, production capacity and vertical specialization on innovation performance in China's electronic manufacturing: Analysis from the supply and demand sides

**DOI:** 10.1371/journal.pone.0200642

**Published:** 2018-07-16

**Authors:** Tong Zhao, Zhijie Song, Tianjiao Li

**Affiliations:** School of Economics and Management, Yanshan University, Qinhuangdao, China; Universidad de Castilla-La Mancha, SPAIN

## Abstract

Manufacturing in China has developed rapidly with the widening and deepening of globalization, but only innovation can industry keep upgrading and enhancing its international competitiveness. This paper combines Global Value Chains (GVCs) and National Value Chains (NVCs) in a unified theory framework, uses Structural Decomposition Analysis (SDA) to explore how innovation capacity, production capacity, and vertical specialization affect innovation performance from the supply and demand sides by examining the case of Electronic Manufacturing of China. We observe that innovation inputs and outputs present strong regional heterogeneity between coastal and inland regions. Although most regions continue to engage in processing trade or assembly manufacturing in GVCs, NVCs are gradually established and led by coastal regions. The results indicate a good chance for cultivating innovation capacity in coastal regions. After assessing the influence of determinants on innovation performance, we observe that from supply-side, innovation capacity has a positive effect on innovation performance, production capacity in coastal regions is improving, and domestic demand for domestic products is increasingly important. From the demand side, innovation capacity continues to have a positive effect on innovation performance, production capacity improves rapidly, and imported intermediate inputs enhance innovation performance more effectively than domestic intermediate inputs.

## Introduction

With the fragmentation of production process, the ICT revolution and falling transportation costs, the productive system (or value chains) has changed worldwide. The production process is “sliced up” into several independent tasks with high value added located in the far upstream and far downstream stages (R&D and marketing), and low value added located in the middle stages (manufacturing and assembling) [1, 2]. To focus on the high value-added stages, low value-added tasks are outsourced to developing countries like China by the leaders (or governors) of Global Value Chains (GVCs). In this manner, developing countries can become involved in GVCs and innovation “followers” through the technology spillover effect [3–5].

China has participated in the systematic division of GVCs since the reform and opening began in 1978 and became a great beneficiary of GVCs since 2001, when China entered World Trade Organization. In a short time, China built-up its production capabilities and became the “world’s factory” with a remarkably large trade surplus, especially in high-tech industries like Electronic Manufacturing [6]. With the refinement of the division of globalization, however, trade in goods in GVCs is gradually moving to trade in tasks[7]. One country or industry can participate in only one or several tasks in GVCs. In this case, China’s significant amount of trade is just a surface phenomenon and cannot represent its international competitiveness; for example, Andreas and Christophe indicated that in GVCs, what you see is not what you get[8], and Xing and Detert decomposed the production value chain of iPhone and found China can only obtain a benefit of less than USD 3.6 for every UDS 100 of iPhones manufactured[9]. China’s industries are located at low value-added stages, such as the manufacturing and assembling stages of GVCs[10, 11].This location is mainly due to the ongoing dependence on technologically sophisticated imported intermediate goods and their associated intellectual property. The original mode of economic growth is unsustainable because of the strengthening of resource and environmental constraints and the increasing cost of labor. Downward pressure on China’s economy has increased and China has entered a “New Normal” state. Prime Minster Li proposed that the key solution is to cultivate a new economic momentum and promote innovation. The engine of development in China must transform from factor-driven to innovation-driven. Thus, how to promote innovation performance and strive to be an innovation-oriented country is critical for China[12].

To improve China’s innovation performance and enhance international competitiveness, some Chinese scholars have suggested that China should take two steps to improve innovation capacity and moves up to high value-added stages in GVCs[13–15]. First, China should establish relatively independent National Value Chains (NVCs). As coastal regions (all China’s coastal regions are in the eastern part of the country) have accumulated a certain level of technology and production experience in GVCs based on their economic and geographical advantages. China should transfer the low value-added tasks undertaken from developed countries and multinational corporations to central and western regions to allow coastal regions to focus on cultivating independent innovation capabilities. The purpose of this transfer is to format NVCs; in which coastal (east) regions engage in high value-added stages such as R&D and marketing, whereas central and western regions engage in low value-added stages. Coastal regions can be the impetus of economic and technological development of central and western regions through technology spillover effects and technology transfer, and realize the overall technological and industrial upgrading of China. The second step is based on the first step. When relatively independent NVCs are established, China can reduce technology dependence on developed countries, gradually get rid of its captured status, and eventually move to high value-added stages in GVCs.

Ample theories and empirical researches have suggested that participating in GVCs can improve innovation performance. Norlela and Paulo found that firms can upgrade innovation performance by exporting[16]. Derick et al. studied iPod and notebook PCs profits in GVCs, and their study suggested that Apple Inc. and its suppliers of key components can get most profits by innovation in GVCs[17]. Pietrobelli and Rabellotti identified that developing countries can improve innovation capability through GVCs[18]. Kafouros et al. found internationalization can improve innovation performance, but it can work only when a firm’s international activity level is beyond a certain threshold[19]. Manufacturing in China can also upgrade its technology through “learning by import” effect, “learning by export” effect and form scale effect to improve international competitiveness [20–23]. However, all the above researches focus on GVCs while ignoring NVCs. As Beverlli et al. pointed out, GVCs should have their domestic foundations[24]. Since there is a large variation in development of economy, resource endowments and government policies across provinces or regions within China, and domestic industries can improve competitiveness in NVCs and be more competitive in GVCs in turn. Regional level perspectives are needed in order to explore the relation between regional linkages and innovation performance.

Furthermore, R&D inputs are the primary engine for wealth creation and direct factor that promote innovation performance, which shows the innovation capability for creating new knowledge [25–27]. As pointed by Kafouros et al., R&D firms with high innovation capacity can develop better products and process, faster, at lower cost and therefore R&D inputs contribute more to a firm’s performance[19]. But we also need to consider production capability as it shows the capability to use and adapt existing knowledge [4]. And Piao et al. suggested that innovation performance of Chinese enterprises is generally stagnating at a low level except a few high-tech industries [28].

The first contribution of this paper is to estimate the impact of innovation capacity, production capacity and vertical specialization in GVCs and NVCs on the innovation performance of China’s electronic manufacturing. This high-tech sector is the industry most deeply involved in GVCs; and must be upgraded through innovation. Many researchers have explored in various aspects of the methods to improve innovation performance and international competitiveness of Manufacturing industries[4, 18, 29–31]; by contrast, this paper attempts to answer this question from another perspective. We focus on innovation capacity, production capacity, and vertical specialization to explore the contribution of each determinant, discover a new explanation of the impact of vertical specialization on innovation performance from the perspectives of innovation and production capacity.

Another contribution of this paper is that we build an innovation performance decomposition model. Most models regarding innovation performance in the literature have used econometric models[28, 32, 33]. Notably, econometric models cannot identify on what level the changes of determinants affect innovation performance. Our model is based on applications of interregional Input-Output (I-O) models and Structural Decomposition Analysis (SDA). The I-O model is a common and popular model in research on GVCs [34–37], and SDA is widely used in researches on employment and the green economy [38–42]; but rarely in innovation research. Our model decomposes the actual change in innovation performance from the supply and demand sides into actual developments in innovation and vertical specialization. More precisely, from the supply side, we decompose the change in innovation performance into the change in innovation capacity, change in production capacity and change in vertical specialization to measure the impact of demand structure from the non home regions. From the demand side, we decompose the change in innovation performance into change in innovation capacity, change in production capacity and change in vertical specialization to measure the impact of domestic intermediate inputs’ competitiveness.

Based on those theoretical concepts and analytical tools, we aim to answer the following questions. What are the innovation inputs and outputs of electronic manufacturing in different regions in China, and what are their changing trends? What are the main determinants of innovation performance? Does the innovation performance of electronic manufacturing benefit from participating in GVCs or NVCs?

The remainder of this paper is structured as follows. Section 2 presents a discussion of the methodology and describes the data issues; Section 3 presents the findings and discussions. Concluding remarks are reported in Section 4.

## Methodology and data

In this section we outline our approach to measure how innovation performance is decomposed into several determinants from the supply and demand sides. In an open economy like China, the distinction among intraregional, domestic, and imported intermediate products and final products is necessary [43]. Thus, for a domestic region s with n industries, region s participates in GVCs and NVCs, and its gross output can be written in the following equations:

From supply-side, we have the equation as follows:

$$X^{s}=L^{ss}\left(Y^{ss}+\sum_{r\neq s}A^{sr}X^{r}+\sum_{r\neq s}Y^{sr}+E^{s}\right)$$ (1)

Where $X^{s}$ is the 1×n column vector of gross output, $L^{ss}\text{=}{\left(I-A^{ss}\right)}^{-1}\cdot X^{s}$ is the n×n local Leontief inverse matrix, $A^{sr}$ is the n×n matrix of interregional inputs coefficients from region s to region r, $Y^{sr}$ is the 1×n column vector of region r’s final demand on products produced in region s, $E^{s}$ is the 1×n column vector of foreign intermediate and final demand on products produced in region s.

From demand-side we have the equation as follows:

$${\left(X^{s}\right)}^{\prime }={\left(X^{s}\right)}^{\prime }H^{ss}+1^{\prime }\cdot \sum_{r\neq s}Z^{rs}+VA^{s}+IM^{s}\text{=}\left(1^{\prime }\cdot \sum_{r\neq s}Z^{rs}+VA^{s}+IM^{s}\right)G^{s}$$ (2)

Where $H^{ss}\text{=}{\left({\hat{X}}^{s}\right)}^{-1}\cdot Z^{ss}$, and $G^{s}\text{=}{\left(I-H^{ss}\right)}^{-1}$ is the n×n local Ghosh inverse matrix. $Z^{sr}$ is the n×n matrix of interregional inputs from region s to region r, $VA^{s}$ is the 1×n row vector of value added generated in region s, $IM^{s}$ is the 1×n row vector of imported intermediate inputs of region s.

### I-O model of innovation performance and decomposition method on the supply side

#### I-O model of innovation performance

Before building the model, we select the appropriate variables to measure innovation input and innovation performance. R&D internal expenditure refers to expenditures for basic research, applied research, and experimental development, including labor costs, raw material costs, construction and acquisition costs, management fees, and other expenses for R&D activities. The model can fully measure the cost of an industry’s R&D activities. R&D intensity (R&D input over gross output) can measure how intensively the industry invests in building and maintaining its knowledge stock[44]. Output value of new products is a widely accepted and commonly used variable representing innovation performance[45–47]. Thus, we use regional R&D internal expenditure and R&D intensity to represent R&D input; and the output value of new products to represent innovation performance for the electronic manufacturing industry in China.

Eq (1) decomposes the gross output of region s based on the intermediate and final demand of different areas. With utilization efficiency vector of R&D input ($\alpha^{s}$) representing the output value of a new product per unit of R&D internal expenditure, and R&D intensity vector ($\beta^{s}$) representing the R&D input per unit of gross output, the output value of the new products of region s ($NP_{s}$) from supply-side can be formulated from Eq (1) as follows:

$$\begin{array}{l} NP_{s}={\hat{\alpha }}^{s}\cdot {\hat{\beta }}^{s}\cdot L^{ss}\cdot \left(Y^{ss}+\sum_{r\neq s}A^{sr}X^{r}+\sum_{r\neq s}Y^{sr}+E^{s}\right)\\ \text{=}\underset{\left(1\right)}{{\hat{\alpha }}^{s}}\cdot \underset{\text{(2)}}{\left(\underset{\left(2a\right)}{{\hat{\beta }}^{s}\cdot L^{ss}\cdot Y^{ss}}+\underset{\left(2b\right)}{{\hat{\beta }}^{s}\cdot L^{ss}\cdot \sum_{r\neq s}A^{sr}X^{r}}+\underset{\left(2c\right)}{{\hat{\beta }}^{s}\cdot L^{ss}\cdot \sum_{r\neq s}Y^{sr}}+\underset{\left(2d\right)}{{\hat{\beta }}^{s}\cdot L^{ss}\cdot E^{s}}\right)} \end{array}$$ (3)

Where a hat (^) indicates a diagonal matrix.

The output value of new products can be decomposed into two main determinants: (1) the utilization efficiency of R&D internal expenditure and (2) total amount of R&D internal expenditure, which can be further decomposed into four parts according to the final or intermediate demand of different areas: (2a) the R&D internal expenditure for final goods demand in the home region, (2b) R&D internal expenditure for intermediate goods demand in other domestic regions, (2c) R&D internal expenditure for final goods demand in other domestic regions and (2d) R&D internal expenditure for foreign demand.

The output value of new products can represent the scale of innovation performance; however, because differences in resources and environment exist among regions, the innovation transformative capacity of different regions are very different in China, and the amount of the output value of new products cannot determine the differences regarding innovation performance among regions. Therefore, we apply the comparable ratio $R^{S}\text{=}NP_{s}/1^{\prime }\cdot X_{s}$ that is, the output value of new products per unit of regional gross output representing innovation performance of region s, which can be formulated as follows:

$$R^{S}=\frac{NP_{s}}{1^{\prime }\cdot X_{s}}=\frac{{\hat{\alpha }}^{s}\cdot {\hat{\beta }}^{s}\cdot L^{ss}\cdot \left(Y^{ss}+\sum_{r\neq s}A^{sr}X^{r}+\sum_{r\neq s}Y^{sr}+E^{s}\right)}{1^{\prime }\cdot X_{s}}\text{=}{\hat{\alpha }}^{s}\cdot {\hat{\beta }}^{s}\cdot L^{ss}\cdot S^{s}$$ (4)

Where $1^{\prime }$ is a 1×n row vector with all elements equal to 1, $S^{s}\text{=}\frac{Y^{ss}+\sum_{r\neq s}A^{sr}X^{r}+\sum_{r\neq s}Y^{sr}+E^{s}}{1^{\prime }\cdot X_{s}}$

#### Decomposition method

SDA has been one of the most popular tools in I-O research and widely applied in the literature on economic, environmental, and social problems [48–52]. We follow the approach adopted by Dietzenbacher and Los, who found that the average of any two mirror decompositions provides a reasonable estimate [53, 54]. This method has been used in SDA analysis [38, 55, 56]. We use the subscripts “1” and “0” to represent the terminal and initial states of a variable during a period of interest, and the form of $\text{C}\left(\right)$ to represent the effects caused by each determinant on innovation performance[57]. According to the methodology, changes in innovation performance of region s ($\Delta R^{S}$) can be decomposed, additively, into the following formula:

$$\begin{array}{l} \Delta R^{S}=\underset{effect\;of\Delta {\hat{\alpha }}^{s}}{\frac{1}{2}\Delta {\hat{\alpha }}^{s}\left({\hat{\beta }}_{1}^{s}L_{1}^{ss}S_{1}^{s}+{\hat{\beta }}_{0}^{s}L_{0}^{ss}S_{0}^{s}\right)⏟}+\underset{effectof\Delta {\hat{\beta }}^{s}}{\frac{1}{2}\left({\hat{\alpha }}_{0}^{s}\Delta {\hat{\beta }}^{s}L_{1}^{ss}S_{1}^{s}+{\hat{\alpha }}_{1}^{s}\Delta {\hat{\beta }}^{s}L_{0}^{ss}S_{0}^{s}\right)⏟}\\ +\underset{effectof\Delta L^{ss}}{\frac{1}{2}\left({\hat{\alpha }}_{0}^{s}{\hat{\beta }}_{0}^{s}\Delta L^{ss}S_{1}^{s}+{\hat{\alpha }}_{1}^{s}{\hat{\beta }}_{1}^{s}\Delta L^{ss}S_{0}^{s}\right)⏟}+\underset{effectof\Delta S^{s}}{\frac{1}{2}\left({\hat{\alpha }}_{0}^{s}{\hat{\beta }}_{0}^{s}L_{0}^{ss}+{\hat{\alpha }}_{1}^{s}{\hat{\beta }}_{1}^{s}L_{1}^{ss}\right)\Delta S^{s}⏟}\\ \text{=C}\left(\Delta {\hat{\alpha }}^{s}\right)+\text{C}\left(\Delta {\hat{\beta }}^{s}\right)+\text{C}\left(\Delta L^{ss}\right)+\text{C}\left(\Delta S^{s}\right) \end{array}$$ (5)

The first stage decomposition attempts to obtain the contribution of four determinants: $\text{C}\left(\Delta {\hat{\alpha }}^{s}\right)$ measures the effect caused by the change in utilization efficiency of R&D internal expenditure and $\text{C}\left(\Delta {\hat{\beta }}^{s}\right)$ measures the effect caused by the change in R&D intensity, and these two determinants are the innovation capacity effect vectors. $\text{C}\left(\Delta L^{ss}\right)$ is the regional production capacity effect from the supply side and $\text{C}\left(\Delta S^{s}\right)$ is the vertical specialization effect in the process of participating in GVCs or NVCs. The last two determinants can be further decomposed into a nested form.

$\text{C}\left(\Delta L^{ss}\right)$ can be further decomposed into two components (the detailed mathematical proof and formula are provided in the S1 File):

$$\text{C}\left(\Delta L^{ss}\right)=\text{C}\left(\Delta A^{s}\right)+\text{C}\left(\Delta s^{Ass}\right)$$ (6)

where $A^{s}\text{=}\sum_{r}A^{sr}$, and $s^{Ass}\text{=}A^{ss}/\sum_{r}A^{sr}$.

In Eq (6), the first term $\text{C}\left(\Delta A^{s}\right)$ is the total intermediate products’ supply proportion component, that is the change in innovation performance due to the change in the totaled intermediate inputs’ coefficients from the home region to all domestic regions, and measures the importance of intermediate products provided by the home region. The second term $\text{C}\left(\Delta s^{Ass}\right)$ is the regional intermediate products’ supply proportion component and measures the change in innovation performance due to the change in the intermediate inputs level for the home region.

$\text{C}\left(\Delta S^{s}\right)$ can be further decomposed into six components (the detailed mathematical proof and formula are provided in the S1 File):

$$C\left(\Delta S^{s}\right)=C\left(\Delta s^{Yss}\right)+\text{C}\left(\Delta r^{A}\right)+\text{C}\left(\Delta \sum_{r\neq s}s^{Asr}\right)+\text{C}\left(\Delta r^{Y}\right)+\text{C}\left(\Delta \sum_{r\neq s}s^{Ysr}\right)\text{+C}\left(\Delta s^{E}\right)$$ (7)

Where $s^{Yss}\text{=}\frac{Y^{ss}}{1^{\prime }\cdot X_{s}}$, $r^{A}\text{=}\frac{1^{\prime }\cdot \sum_{r\neq s}A^{sr}X^{r}}{1^{\prime }\cdot X_{s}}$, $s^{Asr}\text{=}\frac{A^{sr}X^{r}}{1^{\prime }\cdot \sum_{r\neq s}A^{sr}X^{r}}$, $r^{Y}\text{=}\frac{1^{\prime }\cdot \sum_{r\neq s}Y^{sr}}{1^{\prime }\cdot X_{s}}$, $s^{Ysr}\text{=}\frac{Y^{sr}}{1^{\prime }\cdot \sum_{r\neq s}Y^{sr}}$, $s^{E}\text{=}\frac{E^{s}}{1^{\prime }\cdot X_{s}}$

In Eq (7), $\text{C}\left(\Delta S^{s}\right)$ is decomposed into six components. $C\left(\Delta s^{Yss}\right)$ is the level component of final demand by home region and yields the change in innovation performance due to the change in the ratio for the home region’s final demand compared with the regional gross output. $\text{C}\left(\Delta s^{E}\right)$ is the level component of foreign demand and yields the change in innovation performance due to the change in the ratio for exports compared with the regional gross output. $\text{C}\left(\Delta r^{A}\right)$ is the level component of intermediate demand by other domestic regions and yields the change in innovation performance due to the change in the ratio for other domestic regions’ total intermediate demand compared with the regional gross output. $\text{C}\left(\Delta \sum_{r\neq s}s^{Asr}\right)$ is the structural component of intermediate demand by other domestic regions and the total effect of other domestic regions. Each region’s individual effect $\Delta s^{Asr}$ measures the change in innovation performance caused by the change in the ratio for region r’s intermediate demand compared with the total of other domestic regions’ intermediate demand. $\text{C}\left(\Delta r^{Y}\right)$ is the level component of final demand by other domestic regions. $\text{C}\left(\Delta \sum_{r\neq s}s^{Ysr}\right)$ is the structural component of final demand by other domestic regions.

In summary, the change in innovation performance from the supply side can be decomposed into the following 10 determinants:

$$\begin{array}{l} \Delta R^{S}\text{=C}\left(\Delta {\hat{\alpha }}^{s}\right)+\text{C}\left(\Delta {\hat{\beta }}^{s}\right)+\text{C}\left(\Delta A^{s}\right)+\text{C}\left(\Delta s^{Ass}\right)+\text{C}\left(\Delta s^{Yss}\right)\\ +\text{C}\left(\Delta r^{A}\right)+\text{C}\left(\Delta \sum_{r\neq s}s^{Asr}\right)+\text{C}\left(\Delta r^{Y}\right)+\text{C}\left(\Delta \sum_{r\neq s}s^{Ysr}\right)\text{+C}\left(\Delta s^{E}\right) \end{array}$$ (8)

### I-O model of innovation performance and decomposition method on the demand side

#### I-O model of innovation performance

Eq (2) decomposes the gross output of region s based on intermediate demand and value added of the home region. Then the output value of a new product from region s from the demand side can be expressed as follows:

$$\begin{array}{l} NP_{s}={\hat{\alpha }}^{s}\cdot {\hat{\beta }}^{s}\cdot X^{s}={\hat{\alpha }}^{s}\cdot {\hat{\beta }}^{s}\cdot {\left(G^{s}\right)}^{\prime }\cdot \left[{\left(\sum_{r\neq s}Z^{rs}\right)}^{\prime }\cdot 1+{\left(VA^{s}\right)}^{\prime }+{\left(IM^{s}\right)}^{\prime }\right]\\ \text{=}\underset{\left(1\right)}{{\hat{\alpha }}^{s}}\cdot \underset{\text{(2e)}}{\left[\underset{\left(2\right)}{{\hat{\beta }}^{s}\cdot {\left(G^{s}\right)}^{\prime }\cdot {\left(VA^{s}\right)}^{\prime }}+\underset{\text{(2f)}}{{\hat{\beta }}^{s}\cdot {\left(G^{s}\right)}^{\prime }\cdot {\left(\sum_{r\neq s}Z^{rs}\right)}^{\prime }\cdot 1}+\underset{\text{(2g)}}{{\hat{\beta }}^{s}\cdot {\left(G^{s}\right)}^{\prime }\cdot {\left(IM^{s}\right)}^{\prime }}\right]} \end{array}$$ (9)

The output value of new product is also decomposed into two main determinants: (1) the utilization efficiency of the R&D internal expenditure and (2) amount of R&D internal expenditure, which can be further decomposed into three parts from the demand side according to the region participating in GVCs and NVCs: (2e) R&D internal expenditure due to value-added capability of home region, (2f) R&D internal expenditure due to using intermediate inputs from other domestic regions and (2g) R&D internal expenditure due to using import intermediate inputs.

By using the same methodology as the supply-side decomposition, the innovation performance of region s from the demand side will be as follows:

$$R^{D}=\frac{NP_{s}}{1^{\prime }\cdot X_{s}}=\frac{{\hat{\alpha }}^{s}\cdot {\hat{\beta }}^{s}\cdot {\left(G^{s}\right)}^{\prime }\cdot \left[{\left(\sum_{r\neq s}Z^{rs}\right)}^{\prime }\cdot 1+{\left(VA^{s}\right)}^{\prime }+{\left(IM^{s}\right)}^{\prime }\right]}{1^{\prime }\cdot X_{s}}\text{=}{\hat{\alpha }}^{s}\cdot {\hat{\beta }}^{s}\cdot {\left(G^{s}\right)}^{\prime }\cdot D^{s}$$ (10)

Where $D^{s}\text{=}\frac{{\left(\sum_{r\neq s}Z^{rs}\right)}^{\prime }\cdot 1+{\left(VA^{s}\right)}^{\prime }+{\left(IM^{s}\right)}^{\prime }}{1^{\prime }\cdot X_{s}}$

#### Decomposition method

Similarly, the change in innovation performance of region s ($\Delta R^{D}$) can be decomposed, additively, into the following formula:

$$\begin{array}{l} \Delta R^{D}=\frac{\frac{1}{2}\Delta {\hat{\alpha }}^{s}\left[{\hat{\beta }}_{1}^{s}{\left(G_{1}^{s}\right)}^{\prime }D_{1}^{s}+{\hat{\beta }}_{0}^{s}{\left(G_{0}^{s}\right)}^{\prime }D_{0}^{s}\right]}{effectof\Delta {\hat{\alpha }}^{s}}+\frac{\frac{1}{2}\left[{\hat{\alpha }}_{0}^{s}\Delta {\hat{\beta }}^{s}{\left(G_{1}^{s}\right)}^{\prime }D_{1}^{s}+{\hat{\alpha }}_{1}^{s}\Delta {\hat{\beta }}^{s}{\left(G_{0}^{s}\right)}^{\prime }D_{0}^{s}\right]}{effectof\Delta {\hat{\beta }}^{s}}\\ +\frac{\frac{1}{2}\left[{\hat{\alpha }}_{0}^{s}{\hat{\beta }}_{0}^{s}\Delta {\left(G^{s}\right)}^{\prime }D_{1}^{s}+{\hat{\alpha }}_{1}^{s}{\hat{\beta }}_{1}^{s}\Delta {\left(G^{s}\right)}^{\prime }D_{0}^{s}\right]}{effectof\Delta {\left(G^{s}\right)}^{\prime }}+\frac{\frac{1}{2}\left[{\hat{\alpha }}_{0}^{s}{\hat{\beta }}_{0}^{s}{\left(G_{0}^{s}\right)}^{\prime }+{\hat{\alpha }}_{1}^{s}{\hat{\beta }}_{1}^{s}{\left(G_{1}^{s}\right)}^{\prime }\right]\Delta D^{s}}{effectof\Delta D^{s}}\\ \text{=C}\left(\Delta {\hat{\alpha }}^{s}\right)+\text{C}\left(\Delta {\hat{\beta }}^{s}\right)+\text{C}\left[\Delta {\left(G^{s}\right)}^{\prime }\right]+\text{C}\left(\Delta D^{s}\right) \end{array}$$ (11)

Now, the last two terms of Eq (11) must be decomposed.

$\text{C}\left[\Delta {\left(G^{s}\right)}^{\prime }\right]$ can be further decomposed into three components (the detailed mathematical proof and formula are provided in the S1 File):

$$C\left[\Delta {\left(G^{s}\right)}^{\prime }\right]\text{=C}\left[\Delta {\left({\hat{X}}^{s}\right)}^{-1}\right]+\text{C}\left(\Delta Z^{s}\right)+\text{C}\left(\Delta r^{Zss}\right)$$ (12)

where $Z^{s}\text{=}\sum_{r}Z^{rs}$, $r^{Zss}\text{=}Z^{ss}/\sum_{r}Z^{rs}$

In Eq (12), the first term $\text{C}\left[\Delta {\left({\hat{X}}^{s}\right)}^{-1}\right]$ is the total gross output distribution component and measures the change in innovation performance due to the reciprocal of the change in regional output. The second term $\text{C}\left(\Delta Z^{s}\right)$ is the total intermediate demand component of region s is, the change in innovation performance due to the change in the totaled intermediate inputs from all domestic regions to region s. The third term $\text{C}\left(\Delta r^{Zss}\right)$ is the regional intermediate demand component. It is the change in innovation performance due to the change in intermediate inputs level for home region.

$\text{C}\left(\Delta D^{s}\right)$ can be further decomposed into four components (the detailed mathematical proof and formula are provided in the S1 File):

$$\text{C}\left(\Delta D^{s}\right)=\text{C}\left(\Delta r^{Z}\right)+\text{C}\left(\Delta \sum_{r\neq s}s^{Zrs}\right)+\text{C}\left(\Delta s^{V}\right)+\text{C}\left(\Delta s^{I}\right)$$ (13)

Where $r^{Z}\text{=}\frac{1^{\prime }\cdot {\left(\sum_{r\neq s}Z^{rs}\right)}^{\prime }}{1^{\prime }\cdot X^{s}}$, $s^{Zrs}\text{=}\frac{{\left(Z^{rs}\right)}^{\prime }\cdot 1}{1^{\prime }\cdot {\left(\sum_{r\neq s}Z^{rs}\right)}^{\prime }}$, $s^{V}\text{=}\frac{VA_{t}^{s}}{1^{\prime }\cdot X_{s}}$, $s^{I}\text{=}\frac{IM_{t}^{s}}{1^{\prime }\cdot X_{s}}$

In Eq (13), $\text{C}\left(\Delta D^{s}\right)$ is decomposed into four components. $\text{C}\left(\Delta r^{Z}\right)$ is the level component of intermediate demand by region s and yields the change in innovation performance due to the change in the ratio for region s’s total intermediate demand compared with regional gross output. $\text{C}\left(\Delta \sum_{r\neq s}s^{Zrs}\right)$ is the structural component of intermediate demand by region s and represents the total effect of region s’s intermediate demand for other domestic regions. Additionally, each region’s individual effect $s^{Zrs}$ measures the change in innovation performance caused by the change in the ratio of region s’s intermediate demand for region r compared with region s’s total intermediate demand. $\text{C}\left(\Delta s^{V}\right)$ is the level component of the value-added capabilities of the home region. $\text{C}\left(\Delta s^{I}\right)$ is the level component of imported intermediate demand by the home region.

In summary, the change in innovation performance from the demand side can be decomposed into the following 9 determinants:

$$\begin{array}{l} \Delta R^{D}\text{=C}\left(\Delta {\hat{\alpha }}^{s}\right)+\text{C}\left(\Delta {\hat{\beta }}^{s}\right)+\text{C}\left[\Delta {\left({\hat{X}}^{s}\right)}^{-1}\right]+\text{C}\left(\Delta Z^{s}\right)+\text{C}\left(\Delta r^{Zss}\right)\\ +\text{C}\left(\Delta r^{Z}\right)+\text{C}\left(\Delta \sum_{r\neq s}s^{Zrs}\right)+\text{C}\left(\Delta s^{V}\right)+\text{C}\left(\Delta s^{I}\right) \end{array}$$ (14)

### Hierarchies structure of determinants from the supply and demand sides

**Fig 1:**
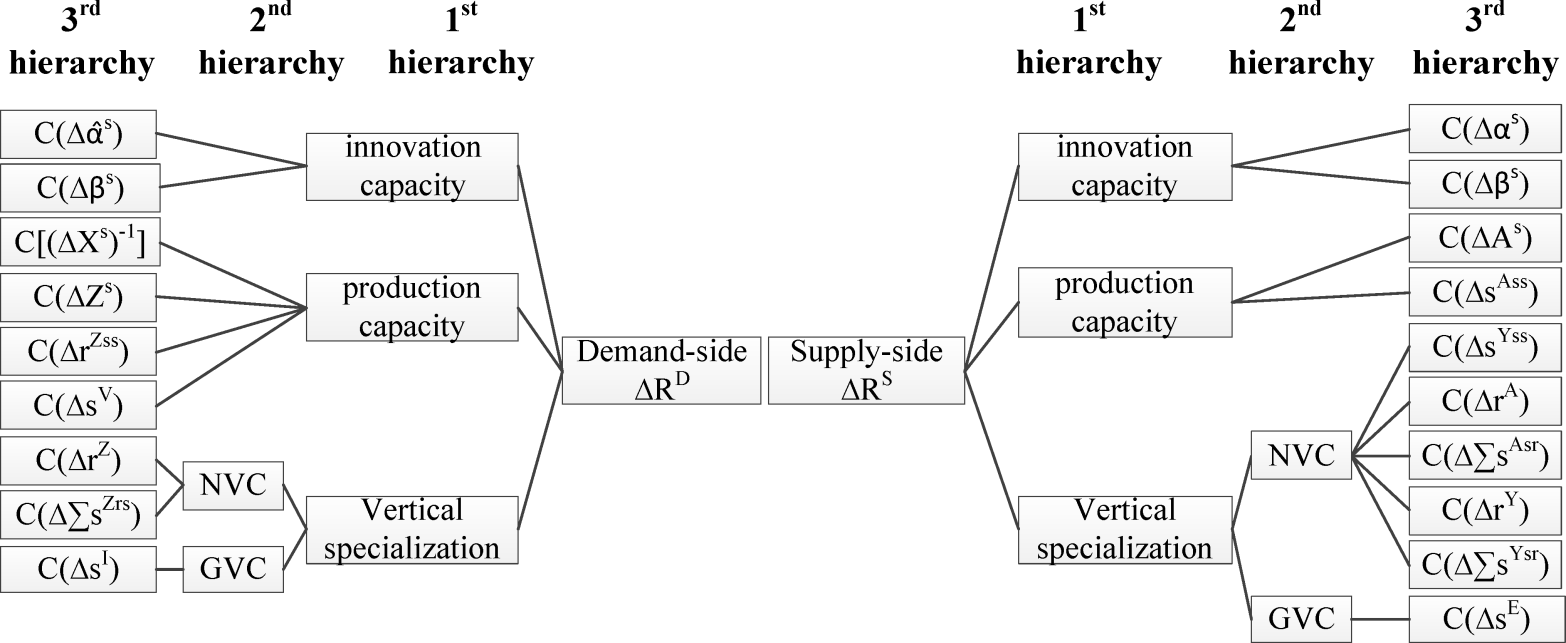
Hierarchical decomposition from the demand and supply sides.

On the basis of 2.1 and 2.2, we classify the effects from supply and demand sides into three hierarchies. We decompose the change in innovation performance into the effect of innovation capacity, production capacity and vertical specialization in the first hierarchy, and further decompose the effect of vertical specialization into the effect of participation in GVCs and NVCs in the second hierarchy. Then at last, we decompose the first and second hierarchies into specific determinants in Eqs (8) and (14) in the third hierarchy. The summarization of determinants is shown in Fig 1.

### Data description

**Fig 2:**
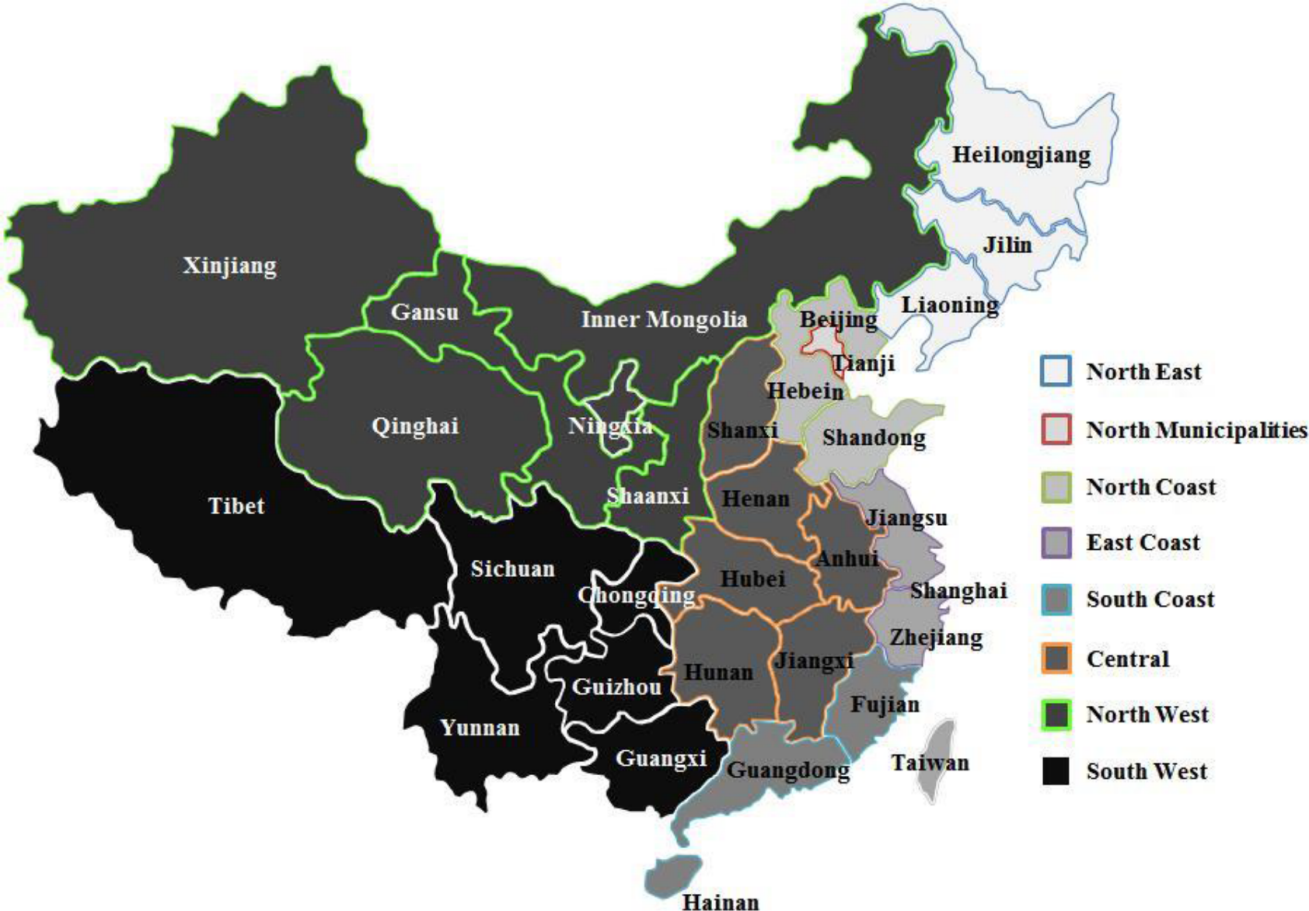
Regional classification of mainland China.

Three databases are used in this study. The first is the 1997, 2002, and 2007 multiregional input-output tables for China compiled by the China State Information Center (SIC) (S2 File), the latest and most complete data on input-output information for eight regions in China. Based on the similarity of economic structures and spatial locations, the 31 provinces are split into eight regions in mainland China (Fig 2). According to the geographical location of the provinces, North Municipalities, North Coast, East Coast, and South Coast are coastal regions, and the other four regions are inland regions.

The R&D internal expenditure of electronic manufacturing is from the 1998–2010 *China Statistics Yearbook on Science and Technology* which contains science and technology statistics from 31 provinces, autonomous regions and municipalities in China compiled by the National Bureau of Statistics and Ministry of Science and Technology of China[58]. The output value of new products from electronic manufacturing is from the 1998–2010 *Technology and China Industry Statistical Yearbook*, which contains industrial economy statistics from 31 provinces, autonomous regions and municipalities in China compiled by the Department of Industry Statistics, National Bureau of Statistics of China[59]. (the data are provided in the S3 File)

Now, we explain several notable points regarding our data:

(1) The input-output tables have a column called “Error”. We split this column into the final demand of different areas (domestic provinces and abroad) according to the final demand structure.
(2) We focus on the electronic manufacturing industry but cannot use the data from this single industry because the $C(\Delta S^{s})$ in Eq (5) and $C(\Delta D^{s})$ in Eq (11) would be zero. Therefore, we use the data from the equipment manufacturing industry with five sub-industries, one of which is the electronic manufacturing industry. The equipment manufacturing industry is the core industry of the manufacturing industry and has capital-, technology-, and labor-intensive characteristics. Technically, there are eight sub-industries according to the *Industrial Classification for national economic activities of China* (GB/T 4754–2017); however, some industries are merged in the input-output tables we used. For example, general equipment manufacturing and special equipment manufacturing are combined to become mechanical industry. Thus, our research on the equipment manufacturing industry uses data from five sub-industries: the metal smelting and products, mechanical, transportation equipment manufacturing, electronic manufacturing, and instrument manufacturing industries. These five sub-industries are the ninth to thirteenth industries in input–output tables; thus, the ${\hat{\alpha }}^{s}$ and ${\hat{\beta }}^{s}$ we apply only have five elements at the position of intersection of ninth to thirteenth columns and ninth to thirteenth rows, and the other elements are all zero. The elements in $X^{s}$ are all zero except the positions of ninth to thirteenth rows.
(3) The input–output tables are presented in current prices, and most research has adjusted them into constant price to compare the results among years [38, 50]. Notably, the indexes in our paper are ratio indexes and eliminate the influence of dimension and price factors; thus, the price factors do not affect the conclusions of this paper.
(4) Considering the time lags from innovation inputs to outputs, we use the current R&D internal expenditure of 1997, 2002, and 2007 as the innovation inputs and output value of new products lag for 1 year as the innovation output.

## Results and discussion

### Trend and decomposition of innovation elements of electronic manufacturing

#### Trend of innovation elements of electronic manufacturing

**Fig 3:**
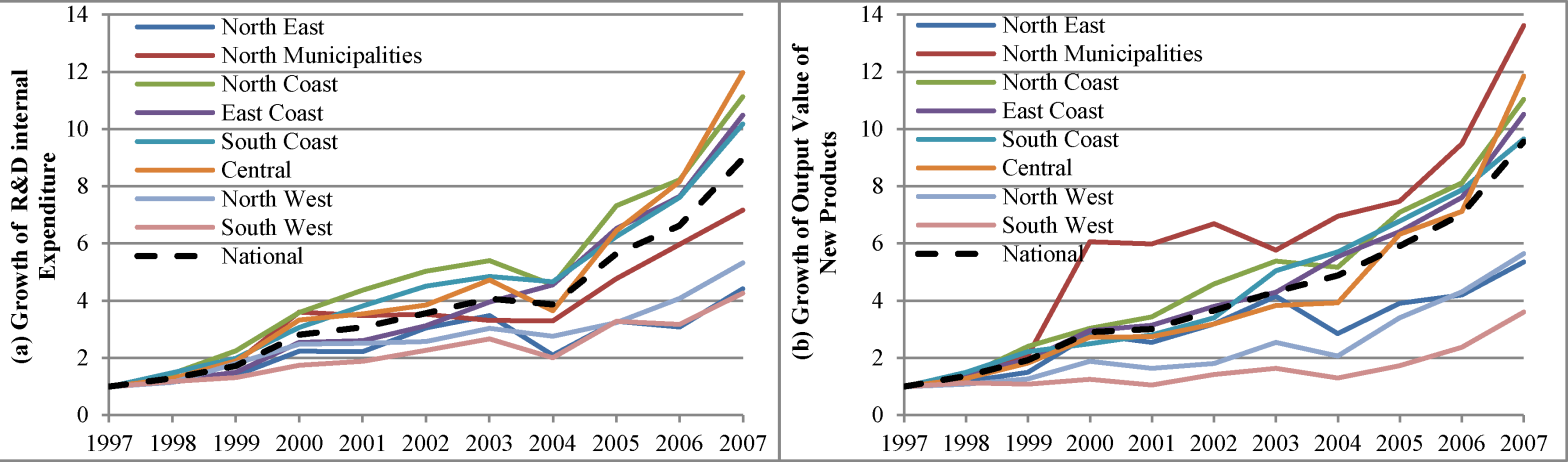
Growth of R&D internal expenditure and output value of new products.

Before the decomposition, we first outline the situation regarding innovation inputs and outputs in the research period from 1997 to 2007. Fig 3 shows the different ratios of the value of two elements in the period 1997–2007 to the value in 1997, representing the growth rate of R&D internal expenditure and output value of new products. At the national average level, the R&D internal expenditure increased 8.9 times, from CNY 0.65 billion in 1997 to CNY 5.81 billion in 2007, and output value of new products increased by 9.6 times, from CNY 6.48 billion in 1997 to CNY 61.89 billion in 2007. The amounts of the two elements are boom through the whole period. The curve of the output value of new products increases faster and more smoothly, and the curve of R&D internal expenditure shows fluctuation growth.

When considering the regional-level growth rate, a variation becomes apparent: the two elements almost increase every year in all regions but at considerably different growth rates.

For R&D internal expenditure in Fig 3A, the growth trends in all regions are almost the same, but coastal regions generally grow faster than inland regions. Four regions have a growth rate of R&D internal expenditure greater than the national average; among which, three are coastal regions and their growth rates in 2007 pass ten times. This result is mainly because China’s developed cities are mostly located in coastal regions. Developed cities have a satisfactory economic foundation and convenient transportation, and their technological advantages have accumulated because of their leadership in participating in GVCs. Therefore, they have a higher demand for technological innovation and require additional investment in innovation.

Notably, Central is a special region with a high R&D internal expenditure growth rate. In 2007, Central became the fastest growing region with its high growth rate, surpassing North Coast. This result is attributed to the “Rise of Central China” plan formulated in 2004. This plan emphasizes the necessity for Central China to focus on the R&D of core and key technologies to develop modern equipment manufacturing and high-tech industries.

Revisiting Fig 3A, we observe that R&D internal expenditure growth rate in Central increases rapidly after 2004. This result implies that under the guidance of national policies, R&D investment in the Central region has increased. The other four regions’ growth rates are less than the national average. North Municipalities comprises Beijing and Tianjin, the economic centers of the Northern China; thus, the growth rates are also relatively high. The other three regions in the North and West fell quite behind than the above regions. From this analysis, it can be observed that the growth rate of R&D internal expenditure shows obvious regional heterogeneity in China.

For the output value of new products in Fig 3B, the situation is somewhat different. The growth rates of three coastal regions and the Central region are still greater than the national average, and North Municipalities takes first place and has the highest growth rates since 2000. This result shows that North Municipalities’ production and innovation capacities are increasing faster than other regions. This rapid increase is due to the political and economic development advantages that Beijing has as the capital of China, and at the same time, the Beijing–Tianjin–Hebei region has been one of the three major economic growth poles in China, which has greatly promoted the development of regional industries.

#### Decomposition of innovation elements of electronic manufacturing

**Fig 4:**
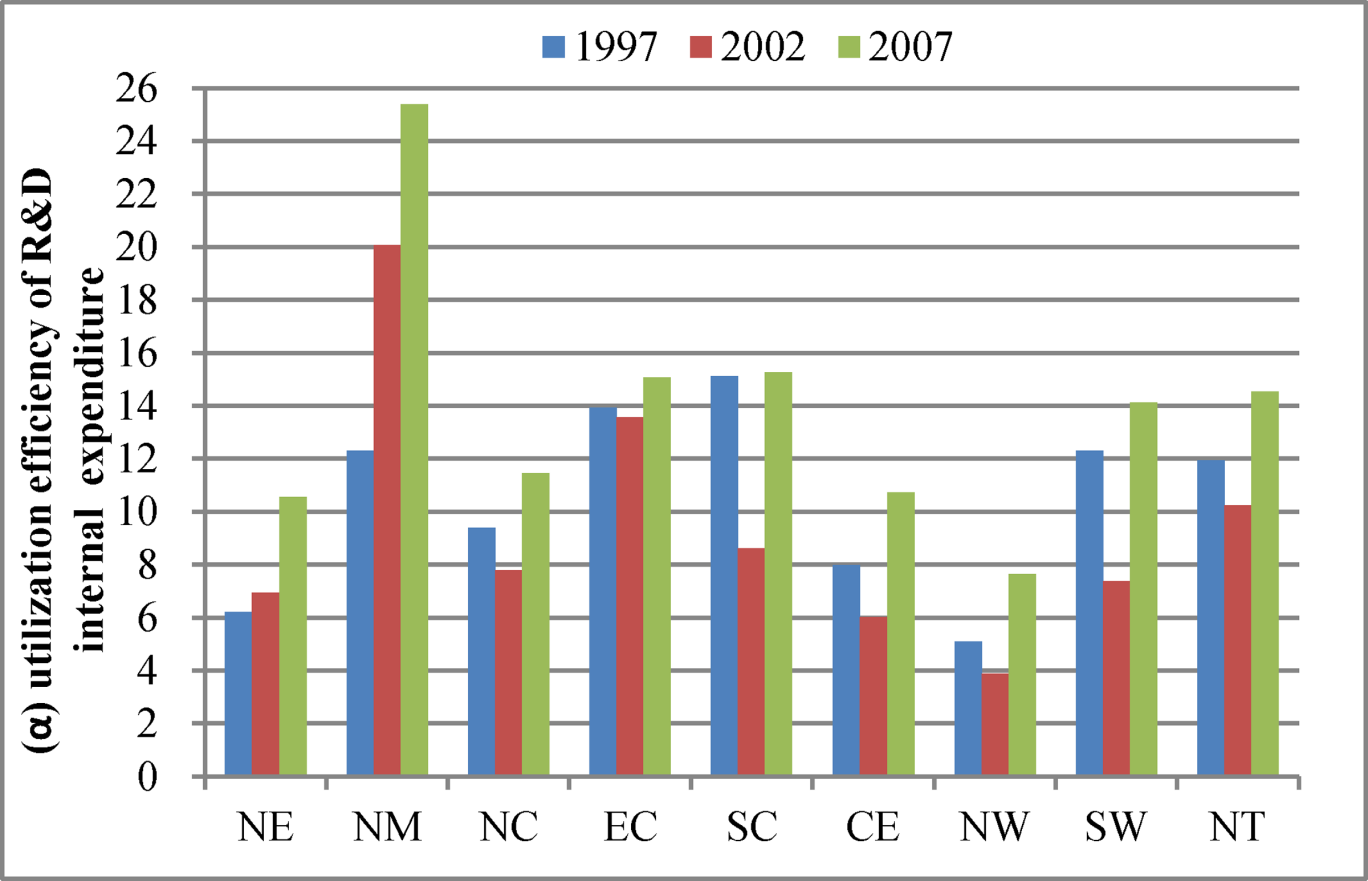
Utilization efficiency of R&D internal expenditure.

**Fig 5:**
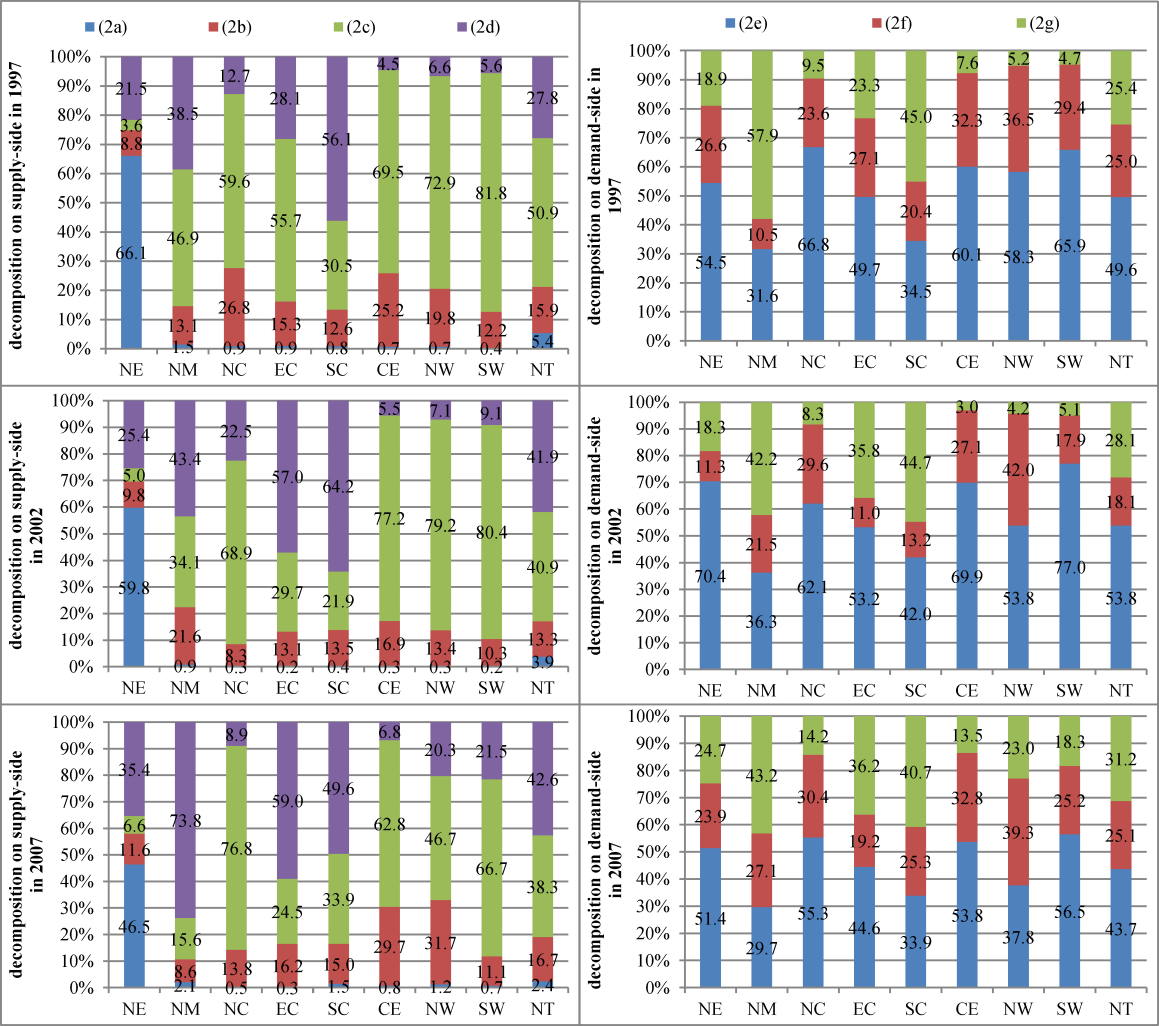
Decomposition of R&D internal expenditure from the supply and demand sides. Abbreviations are used for each region, and “NT” means national level.

After analyzing the growth trends of innovation elements, we must recognize the main determinants that affect innovation performance. We decompose the output value of new products from the supply and demand sides according to Eqs (3) and (9). We obtain two main determinants from the supply and demand sides: (1) the utilization efficiency of R&D internal expenditure Figs 4 and 2 amount of R&D internal expenditure, which can be further decomposed into several parts linked to interregional trade and international trade (Fig 5).

In Fig 4, the utilization efficiency of R&D internal expenditure fluctuates from 1997 to 2007 in all regions. The North Municipalities occupy the first place in 2007 because of the rapid increase in output value of new products. North Municipalities and North East are the only two regions whose utilization efficiencies of R&D internal expenditure increase throughout the period. The ratios in the other six regions deteriorate at some level in 2002 and recover or increase in 2007. Additionally, we observe that although growth rates of R&D internal expenditure and output value of new products in Central are high, Central has relatively low utilization efficiency of R&D internal expenditure. This result shows that industrial development cannot focus only on the amount of R&D inputs, but must also improve the innovation capacity to enhance the utilization efficiency of R&D inputs.

Fig 5 shows the decomposition results of R&D internal expenditure. We can obtain the main determinants and their changing trends that affect R&D internal expenditure. From the supply side decomposition results, the main contributor is R&D internal expenditure for the final demand of other domestic regions (2c) and accounts for 50.9% of R&D internal expenditure at national level in 1997; however, as the years roll on, the share shrinks to 38.3% in 2007 and is replaced by R&D internal expenditure for foreign demand (2d), which increases from 27.8% to 42.6% and becomes the most important determinant. At the regional level, we observe that R&D internal expenditure for final demand (2a, 2c) decreases in most regions; as a result, R&D internal expenditure for foreign demand (2d) increases at breakneck speed. R&D internal expenditure for intermediate demand in other domestic regions (2b) becomes more important in the inland regions. From the demand side, R&D internal expenditure for using domestic and imported intermediate inputs (2f, 2g) gradually replaces R&D internal expenditure caused by value-added capability of the home region (2e).

By combing Figs 4 and 5, we can obtain some preliminary characteristics of Chinese electronic manufacturing in GVCs and NVCs:

(1) North Municipalities has the highest ratio of utilization efficiency of R&D internal expenditure; its R&D internal expenditure for foreign demand increases sharply from 38.5% to 73.8% on the supply side; its R&D internal expenditure, due to using domestic intermediate inputs, increases; its R&D internal expenditure, due to using imported intermediate inputs, slightly decreases but still accounts for 43.2% in 2007 from the demand side. These results mean that North Municipalities is mainly engaged in processing exports, and its high utilization efficiency of R&D internal expenditure may because of the technology spillover effects of imported intermediate inputs. This phenomenon may also exist in East Coast and South Coast and indicates that although developed regions in China are still mainly engaged in processing trade in GVCs and have high degree of dependence regarding foreign technology and sophisticated intermediate products, the NVCs have also been gradually established.
(2) North Coast is different from other coastal regions. Although its R&D internal expenditure growth rates and output value of new products are not much different from other coastal regions, its utilization efficiency of R&D internal expenditure is sixth out of eight regions. When checking the structure of R&D internal expenditure, we observe that the amount for domestic final demand is extremely high from the supply side and R&D internal expenditure, due to using intermediate goods, increases from the demand side. This result indicates that North Coast is basically engaged in assembly manufacturing, and its final products are mainly for the domestic market, which is the sign for “low-end lock” in GVCs.
(3) Central, North West, and South West are relatively backward inland regions in China and show the same characters in GVCs and NVCs. From the supply side, R&D internal expenditure for final demand in other domestic regions is the main contributor in 1997 and 2002, and R&D internal expenditure for intermediate demand gradually increases in 2007. From the demand side, R&D internal expenditure due to use of imported intermediate inputs increases and indicates that inland regions are gradually participating in GVCs and NVCs. By participating in GVCs, inland regions can undertake tasks from developed countries due to their abundant labor force and natural resources endowment; thus, they can promote the technology and development of a regional economy through technology spillover effects. By participating in NVCs, inland regions can undertake tasks from coastal regions, and coastal regions can focus on high value-added stages of GVCs and NVCs.
(4) North East is a special region. Its R&D internal expenditure is mostly used for the regional final demand and accounts for almost 50%, even in 2007. Although the ratio decreases by 19.6% from 1997 to 2007, the decrease is basically replaced by foreign demand. From the demand side, R&D internal expenditure due to using imported intermediate inputs increases, and R&D internal expenditure due to using domestic intermediate inputs decreases slightly. We can conclude that East Coast hardly participated in NVCs from the supply side and involved in GVCs.

The deepening and expanding of international production division have greatly promoted the development of international trade in intermediate products and increased the proximity and frequency of interregional and international linkages. The impact of intermediate products trade on innovation is becoming increasingly important. But because China’s technology lags behind that of developed countries, the products are short of international competitiveness; thus, China’s industries must undertake the low-end tasks outsourced by developed countries or depend on sophisticated, imported intermediate inputs to participate in GVCs. Notably, the profit through these means is relatively low. Industries must continue to innovate to enhance the competitive advantage in international competition.

### SDA of the change in innovation performance on the supply side

To recognize the influence of different determinants on innovation performance, we decompose the change in innovation performance on the supply and demand sides based on Fig 1 and discuss the results hierarchically.

#### Decomposition results on first hierarchy

**Fig 6:**
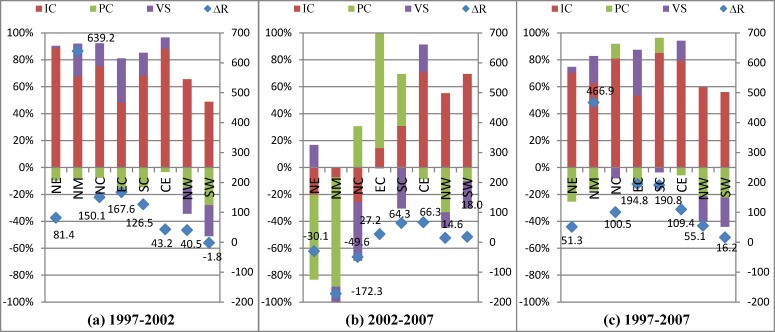
Decomposition of the first hierarchy from the supply side (%). Legends IC, PC, and VS are abbreviations of innovation capacity, production capacity, and vertical specialization. Scatter diagram ΔR shows the results of innovation performance change multiplied by 10,000, which means the increased amount of output value of new products if total output increases by 10000 units. The percentage stacking bar charts show the contribution of each decomposition item and represent the percentages of each decomposition factor in the change in innovation performance and accumulated together.

According to Fig 1, we can decompose the change in innovation performance in the first hierarchy into three parts: the change in innovation capacity, the change in production capacity and the change in vertical specialization. The influences of the three factors have considerable differences in different periods (Fig 6).

From 1997 to 2002, innovation performance increases in all regions except South West. North Municipalities is the fastest growing region and its increase in innovation performance reaches is nearly four times that of the second fastest region, East Coast. Innovation capacity is the main contributor and can improve innovation performance by an average of 69%. Vertical specialization has a negative contribution in North West and South West, approximately 20%, and has a positive contribution in the other six regions: the highest and lowest are 32.47% and 1.36% for East Coast and North East. Production capacity has a negative contribution in all regions and ranges from -3.39% to -27.96%. This range shows that the promotion of its innovation capacity and its active participation in GVCs and NVCs as a supplier have greatly improved the innovation performance of Electronic Manufacturing in China, but the short of production capacity still restricts the development of the industry.

In the period 2002–2007, the situation is different. The innovation performance trend is almost the opposite of that from 1997–2002, and the change in innovation performance decreases by approximately 30% in North East, North Municipalities, and North Coast. Innovation capacity is the main positive contributor in Central and West, and production capacity is the main contributor in most regions with positive and negative effects. Vertical specialization has a positive effect in only North East and Central. Because of the deepening of globalization, China’s regional development is becoming more unbalanced. Production capacity increases in coastal regions, except North Municipalities, and becomes the greatest hindrance to promoting innovation performance in the inland regions. Insufficient innovation capacity and weaker domestic and foreign demand causes poor innovation performance in the industry.

#### Decomposition results for the second and third hierarchies

We can obtain information from the analysis of the results from the first hierarchy; however, we can observe only three factors that influence innovation performance. Thus, we report the influence of factors in the second and third hierarchies in Table 1 to obtain additional details about the three factors.

**Table 1:** Decomposition of the second and third hierarchies from the demand side (%).

		Innovation Capacity		Production Capacity		NVCs					GVCs
		$\text{C(}\Delta {\hat{\alpha }}^{s}\text{)}$	$\text{C(}\Delta {\hat{\beta }}^{s}\text{)}$	$\text{C(}\Delta A^{s}\text{)}$	$\text{C(}\Delta s^{Ass}\text{)}$	$\text{C(}\Delta s^{Yss}\text{)}$	$\text{C(}\Delta r^{A}\text{)}$	$\text{C(}\Delta \sum s^{Asr}\text{)}$	$\text{C(}\Delta r^{Y}\text{)}$	$\text{C(}\Delta \sum s^{Ysr}\text{)}$	$\text{C(}\Delta s^{E}\text{)}$
1997–2002	NE	7.30	58.17	-5.88	-1.15	-3.22	4.80	-3.49	7.42	-6.54	2.03
	NM	27.38	39.72	-3.32	-4.58	-0.43	1.90	7.07	2.18	1.60	11.83
	NC	-9.96	54.15	-7.47	2.97	11.19	-6.22	0.37	-0.60	-0.99	6.09
	EC	-2.06	44.41	-9.49	-6.91	-2.61	0.60	1.30	-0.18	-1.65	30.79
	SC	-29.16	51.35	0.55	-5.32	-2.90	2.24	-1.13	0.85	-0.01	6.49
	CE	-19.19	52.85	-3.21	1.92	8.59	-7.66	3.63	-0.67	-1.51	0.78
	NW	-15.75	55.99	-11.36	2.18	-0.78	-6.55	1.39	-3.39	-2.03	-0.58
	SW	31.45	45.18	-8.18	0.33	-4.39	0.50	-1.65	-4.63	2.00	1.69
2002–2007	NE	29.51	-34.62	-14.30	-1.58	-5.15	-0.31	2.21	-2.42	3.80	6.09
	NM	15.87	-17.02	-15.51	2.67	-10.66	2.61	-11.43	3.06	-3.27	17.89
	NC	24.17	-33.89	13.26	-1.67	-6.25	4.37	-3.50	-1.22	0.92	-10.76
	EC	22.58	-20.00	13.02	2.05	-13.50	-2.91	9.82	-4.82	4.78	6.53
	SC	37.22	-27.97	5.48	6.04	3.69	1.43	-2.08	1.96	-0.39	-13.74
	CE	43.77	-10.94	4.57	-8.57	-11.32	6.84	4.88	1.31	6.33	1.47
	NW	33.53	-8.62	-10.04	-4.95	-22.84	8.16	-1.20	5.59	0.25	4.83
	SW	30.25	-22.48	-0.90	-0.22	-9.61	2.02	-1.77	15.02	-12.84	4.89
1997–2007	NE	29.58	24.77	-17.28	-2.19	-8.23	4.06	-1.24	3.74	-1.99	6.93
	NM	27.70	20.94	-12.63	-0.65	-8.54	1.91	-1.85	3.23	-0.83	21.72
	NC	15.84	46.53	4.45	3.96	11.41	-3.45	-7.04	-3.47	0.12	-3.75
	EC	5.78	36.17	-3.49	-6.35	-8.22	-0.38	4.75	-2.31	0.50	32.04
	SC	0.89	57.34	11.40	-3.75	-1.11	7.14	-5.51	4.93	-0.65	-7.28
	CE	21.66	46.79	1.09	-6.11	-2.21	-3.61	11.12	0.35	4.84	2.21
	NW	14.92	36.62	-15.56	-2.84	-20.23	1.24	-0.10	2.51	-2.97	3.01
	SW	8.95	18.20	-11.49	0.67	-18.15	4.03	-5.18	11.94	-12.29	9.12

Columns show the contribution of each decomposition item, namely, the percentages of each decomposition determinant in the change in innovation performance.

When we examine the two factors in innovation capacity, we observe that although utilization efficiency of R&D internal expenditure ($\text{C(}\Delta {\hat{\alpha }}^{s}\text{)}$) and R&D intensity ($\text{C(}\Delta {\hat{\beta }}^{s}\text{)}$) have positive effects for the duration of 1997–2007, $\text{C(}\Delta {\hat{\alpha }}^{s}\text{)}$ inhibits the innovation performance from 1997–2002 and becomes the main impetus promoting innovation performance from 2002–2007. The situation of $\text{C(}\Delta {\hat{\beta }}^{s}\text{)}$ is the opposite of $\text{C(}\Delta {\hat{\alpha }}^{s}\text{)}$ and indicates that from 1997–2002, the electronic manufacturing industry in China is mainly committed to increasing R&D investment. However, due to the limited industrial technology capabilities, the industry cannot absorb excessive R&D investment to transform into innovation output. Therefore, R&D intensity has greater role in promoting innovation performance but insufficient utilization of R&D restricts innovation performance. From 2002–2007, the electronic manufacturing industry in China had accumulated a certain degree of R&D investment and begun to shift to improving the technology and R&D level of the industry. Thus, the utilization efficiency of R&D internal expenditure at this stage has a strong promotion effect on innovation performance.

For production capacity, the total intermediate products supply proportion component ($\text{C(}\Delta A^{s}\text{)}$) is the main contributor. This component has a negative effect in most regions from 1997–2002, and gets worse in inland regions and better in coastal regions from 2002–2007. Regional intermediate products supply proportion ($\text{C(}\Delta s^{Ass}\text{)}$) improves through the periods in most coastal regions. This result means that production capacity of intermediate products in coastal regions improves, and intermediate products produced by coastal regions become more important in domestic market.

We analyze the effect of vertical specialization through two aspects of regional participation in GVCs and NVCs. In GVCs, we observe that exports have a great role in promoting innovation performance in two periods. The exceptions are North Coast and South Coast, whose innovation performance decreased by 10.7% and 13.7%, respectively, from 2002–2007. In Fig 5, we observe that the two regions have turned to the domestic market for final goods, showing that coastal regions have gradually shifted from GVCs to NVCs, which is the first step in building relatively independent NVCs in China and fostering innovation capacity in coastal regions.

In NVCs, the final demand for home region has a negative effect because most regions participate in GVCs and NVCs. Other domestic regions’ intermediate and final demands ($\text{C(}\Delta r^{A}\text{)}$ and $\text{C(}\Delta r^{Y}\text{)}$) become increasingly important. Since $\text{C(}\Delta \sum s^{Asr}\text{)}$ and $\text{C(}\Delta \sum s^{Ysr}\text{)}$ are the sum of the influence of each region, Table 2 reports the structure of intermediate and final demand of other domestic regions. We observe that the effects of intermediate demand change from positive to negative in coastal regions. This result may reflect the effect of using imported intermediate inputs. The effects of final demand in inland regions changes from negative to positive, especially the demand from coastal regions to inland regions, and this result shows the improvement in the domestic competitiveness of products produced in coastal regions.

**Table 2:** Intermediate and final demand structures from the supply side (%).

		Intermediate demand		Final demand	
		Coastal regions	Inland regions	Coastal regions	Inland regions
1997–2002	NE	-52.75	-47.25	-47.06	-52.94
	NM	99.35	-0.65	76.02	-23.98
	NC	54.83	-45.17	23.66	-76.34
	EC	72.12	-27.88	-3.63	-96.37
	SC	-45.28	-54.72	-53.49	46.51
	CE	93.78	-6.22	-11.92	-88.08
	NW	61.47	-38.53	14.18	-85.82
	SW	-7.55	-92.45	65.95	34.05
2002–2007	NE	69.37	30.63	49.32	50.68
	NM	-81.98	-18.02	-99.09	0.91
	NC	-89.02	-10.98	-15.03	84.97
	EC	96.13	-3.87	62.99	37.01
	SC	-45.63	-54.37	-61.60	38.40
	CE	89.72	-10.28	85.69	14.31
	NW	-79.28	20.72	87.81	12.19
	SW	-52.17	-47.83	-48.85	-51.15

### SDA of the change in innovation performance on the demand side

In Section 3.2, we discussed the effects of various factors on innovation performance on the supply side. Next, we report the results on the demand side.

#### Decomposition results for the first hierarchy

**Fig 7:**
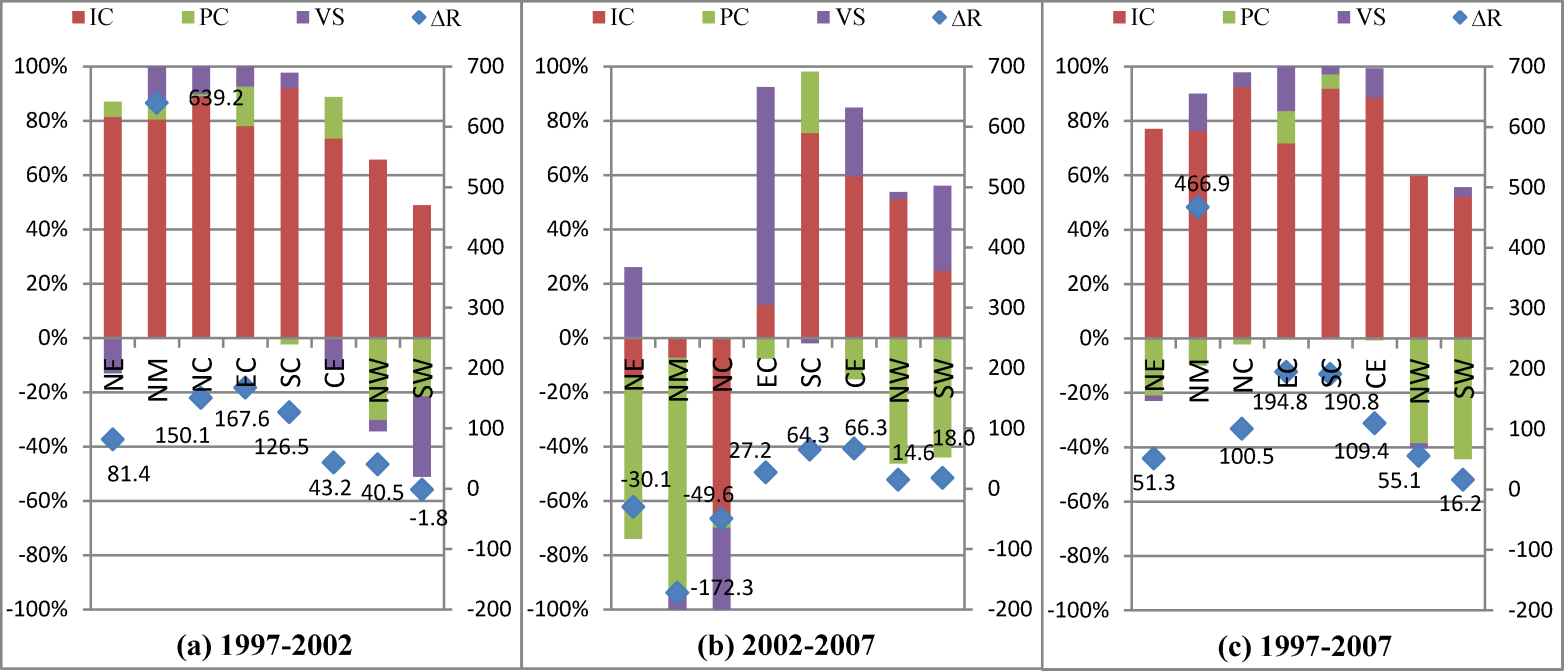
Decomposition of the first hierarchy from the supply side (%).

We decompose the change in innovation performance into three factors on the demand side (Fig 7). Because the trend of innovation capacity is the same as the demand side, we focus on the influence of the last two factors.

Comparing the two factors (PC and VS) in Fig 7, we observe that production capacity has a positive effect in most regions from 1997–2002 but changes into negative effects in all regions except South Coast from 2002–2007. Production capacity decreases innovation performance in inland regions and North Municipalities outwardly; but is not actually the case. We must clarify the specific impact of production capacity in further decomposition results. Vertical specialization shows positive effects only in coastal regions from 1997–2002 and then has positive effects in most regions from 2002–2007, showing the benefit of participating in GVCs and NVCs.

#### Decomposition results for the second and third hierarchies

Table 3 shows the specific decomposition results for the second and third hierarchies. Here, we also focus on the impact of the factors except those in the category of innovation capacity.

**Table 3:** Decomposition of the second and third hierarchies from the demand side (%).

		Innovation Capacity		Production Capacity				NVCs		GVCs
		$\text{C(}\Delta {\hat{\alpha }}^{s}\text{)}$	$\text{C(}\Delta {\hat{\beta }}^{s}\text{)}$	$\text{C(}\Delta {\text{(}X^{s}\text{)}}^{-1}\text{)}$	$\text{C(}\Delta Z^{s}\text{)}$	$\text{C(}\Delta r^{Zss}\text{)}$	$\text{C(}\Delta s^{V}\text{)}$	$\text{C(}\Delta r^{Z}\text{)}$	$\text{C(}\Delta s^{Zrs}\text{)}$	$\text{C(}\Delta s^{I}\text{)}$
1997–2002	NE	5.60	44.58	-19.21	5.71	5.73	11.17	-5.56	-1.84	-0.61
	NM	19.88	28.84	-14.16	14.44	-5.58	9.01	5.10	2.25	0.74
	NC	-7.88	42.85	-19.97	21.12	-2.49	1.58	3.94	0.11	-0.05
	EC	-1.30	28.10	-24.22	17.13	4.74	7.35	-7.33	1.32	8.52
	SC	-23.73	41.78	-14.75	7.49	0.02	6.77	-2.18	0.82	2.46
	CE	-11.56	31.83	-22.78	19.45	0.77	6.78	-3.28	1.86	-1.69
	NW	-14.06	49.96	-20.63	7.32	-3.44	0.13	1.12	-2.30	-1.05
	SW	-20.92	30.05	-23.78	16.08	0.65	3.02	-5.08	-0.35	-0.07
2002–2007	NE	12.00	-14.06	-28.27	30.90	-3.37	-7.70	2.67	0.05	0.98
	NM	10.78	-11.68	-32.99	32.08	-2.12	-6.41	1.72	-0.72	-1.49
	NC	6.29	-9.10	-37.34	39.42	1.88	-4.02	-0.31	-1.26	0.37
	EC	2.96	-2.66	-40.05	45.37	-1.50	-4.02	2.77	-0.63	0.03
	SC	16.55	-12.67	-25.90	32.26	-0.98	-4.21	3.66	-1.01	-2.75
	CE	14.62	-3.78	-32.66	35.09	-1.71	-3.50	3.90	-2.01	2.73
	NW	22.18	-5.86	-29.25	24.47	-2.38	-6.97	-0.93	-3.14	4.83
	SW	14.87	-11.37	-30.44	30.44	-1.67	-4.15	2.90	-1.47	2.70
1997–2007	NE	10.70	8.96	-39.43	36.60	-1.10	-1.44	-0.07	-1.05	0.64
	NM	15.15	11.45	-31.55	30.97	-4.41	1.52	3.78	1.08	-0.10
	NC	2.05	6.03	-42.73	44.67	0.20	-2.32	0.95	-0.76	0.28
	EC	1.27	7.96	-42.76	43.60	0.59	0.11	-0.77	-0.04	2.90
	SC	0.21	13.30	-40.02	41.71	-1.41	0.50	1.64	-0.42	-0.79
	CE	4.76	10.27	-40.25	40.59	-0.84	0.38	1.20	-0.55	1.15
	NW	6.80	16.69	-36.15	28.18	-2.80	-4.44	-0.12	-2.71	2.11
	SW	2.57	5.24	-44.21	40.57	-0.56	-2.45	0.31	-1.95	2.14

The columns show the contribution of each decomposition item, namely, the percentages of each decomposition determinant in the change in innovation performance.

We observe that the total gross output distribution component ($\text{C(}\Delta {\left(X^{s}\right)}^{-1}\text{)}$) and total intermediate demand component ($\text{C(}\Delta Z^{s}\text{)}$) are the main contributors to production capacity. $\text{C(}\Delta {\left(X^{s}\right)}^{-1}\text{)}$ shows a negative effect; however, the greater the amount of total gross output, the smaller the reciprocal of total gross output; thus, the negative results and importance of the factor indicates the rapid growth of total gross output. This phenomenon is the manifestation of China’s rapid economic development since 1978. $\text{C(}\Delta Z^{s}\text{)}$ shows an increasing positive effect, meaning the increment of using domestic intermediate inputs has improved the innovation performance. The regional intermediate demand component ($\text{C(}\Delta r^{Zss}\text{)}$) and the level component of value-added capabilities of home region ($\text{C(}\Delta s^{V}\text{)}$) have little impact on innovation performance, but both worsen through the research periods and inhibit the growth of innovation performance.

For vertical specialization, the impact of participation in GVCs and NVCs are not important, compared with the other two first-hierarchical factors, but we can obtain information about how China’s electronic manufacturing industry participates in GVCs and NVCs from the demand side. Table 4 reports the structure of three determinants in the vertical specialization category. The main effect of domestic intermediate inputs is from coastal regions and changes from positive to negative through the research periods. The effect of imported intermediate dominates three determinants by a margin of 53.1% to 99.2% and plays a positive role in most regions. Additionally, the absolute value of all the proportions are greater than 84%, except East Coast. These results prove that imported intermediate inputs can enhance the innovation performance more effectively than domestic intermediate inputs.

**Table 4:** Intermediate demand structure from the demand side of production (%).

	1997–2002			2002–2007		
	Coastal regions	Inland regions	Abroad	Coastal regions	Inland regions	Abroad
NE	-16.01	-3.76	-80.24	-0.37	0.50	-99.13
NM	54.65	11.31	34.04	-0.15	-7.62	92.23
NC	40.18	-31.23	-28.59	-12.11	-3.11	-84.78
EC	0.54	1.98	97.48	-39.24	7.67	53.08
SC	3.66	0.38	95.96	-1.79	-0.42	-97.79
CE	6.19	-1.80	-92.01	-4.98	0.40	94.62
NW	-4.65	-3.55	-91.81	-0.31	-0.59	99.11
SW	0.06	-0.90	99.04	-0.74	-0.09	99.18

Coastal regions and inland regions represent the impact of participation in NVCs, and “abroad”, the impact of participation in GVCs.

## Conclusion

China has experienced rapid growth and rapidly increased its participation in GVCs since the reform and opening in 1978; however, this growth basically relies on an abundant low–cost labor force and natural resources. As a result of the disappearance of the demographic dividend and deterioration of the ecological environment, Manufacturing (especially high-tech Manufacturing) in China must manage the predicament of “low-end lock” in GVCs. How to move up to high value-added stages in GVCs through innovation is critical for manufacturing in China.

To elucidate the situation of innovation performance and effect of determinants on innovation performance in manufacturing in China, we examined the electronic manufacturing industry from 1997–2007 as an example, built a model of innovation performance based on the input-output model, and combined GVCs and NVCs in a unified framework. The model focused on three categories of determinants, namely, innovation capacity, production capacity, and vertical specialization, and analyzed the impact of determinants on innovation performance using SDA regarding two aspects: the supply and demand sides.

The main conclusions can be summarized as follows:

(1) Innovation inputs and outputs increase at the national level, but variation exists between coastal and inland regions. The innovation elements of coastal regions increase much faster than inland regions.
(2) The utilization efficiency of R&D internal expenditure in most regions first decreases and then increases during the two periods: 1997–2002 and 2002–2007. From the supply side, most R&D internal expenditure is used in the final demand of other domestic regions and foreign demand. From the demand side, R&D internal expenditure for domestic and imported intermediate inputs gradually replaces R&D internal expenditure for regional value-added capacity.
(3) For the effect of the determinants on innovation performance from the supply side, innovation capacity is the most important determinant and promotes the innovation performance throughout the period; and production capacity of intermediate goods in coastal regions improves, which shows that coastal regions have become increasingly important in NVCs. The situation of vertical specialization shows that NVCs are becoming increasingly important, and the competitiveness of the final products of coastal regions increase.
(4) For the effect of determinants on innovation performance from the demand side, innovation capacity remains a critical determinant for the entire period; and production capacity is the most important determinant, and this result indicates the rapid development of industry and increasing importance of domestic intermediate inputs. The impact of vertical specialization is not notable, but its structure shows that imported inputs continue to occupy the absolute proportion, indicating that imported inputs enhance the innovation performance more effectively than domestic intermediate inputs.

The limitations of this study must be mentioned. (1) The upgrading of manufacturing is important for the China’s development, especially upgrading through innovation. This issue is not exclusive to high-tech manufacturing. Due to data limitations, there is no officially published database report for innovation data related to all manufacturing industries; thus, the conclusions in this paper may not coincide with other manufacturing industries. (2) The special feature of processing trade and ownership of the company for manufacturing in China is definitely important and many studies have examined their impact on China’s participation in GVCs [60–63]. However, due to data limitations, we could not obtain detailed data on processing trade of electronic manufacturing or data on enterprises with different ownerships. This limitation may cause bias. A firm-based database or survey would help to further explore the impact of processing trade and firm’s ownership on innovation performance.

## Supporting information

S1 File The mathematical proof and formula detail of Eqs (6), (7), (12) and (13). (DOCX)

S2 File Interregional input-output tables of China in 1997, 2002, 2007. (XLS)

S3 File R&D internal expenditure and output value of new products of China. (XLS)
