# Supplementary material for: Effect of innovation capacity, production capacity and vertical specialization on innovation performance in China's electronic manufacturing: Analysis from the supply and demand sides
Source: PLoS One. 2018 Jul 16;13(7):e0200642. doi: 10.1371/journal.pone.0200642 (PMC6065606; doi:10.1371/journal.pone.0200642)
Supplement: S1 File — (DOCX) [file pone.0200642.s001.docx]

# Appendix A: Derivation of Equation (6)

For $\text{C}\left(\Delta L^{ss}\right)$, recall by definition, we have

$\Delta L^{ss}=L_{1}^{ss}-L_{0}^{ss}=L_{1}^{ss}\Delta A^{ss}L_{0}^{ss}$ (A1)

We define “#” as element-wise matrix multiplication operation, which means if a $n\times n$ matrix is multiplied by another $n\times n$ matrix, the elements in the first matrix is multiplied by the corresponding elements in the second matrix, and we define “/” as the element-wise matrix division operation. We have the following formula from Eq.(A1) as:

$\Delta L^{ss}=L_{1}^{ss}-L_{0}^{ss}=L_{1}^{ss}\Delta A^{ss}L_{0}^{ss}\text{=}L_{1}^{ss}\Delta\left(A^{s}\text{\#}s_{a}^{ss}\right)L_{0}^{ss}$ (A2)

where $A^{s}\text{=}\sum_{r} A^{sr}$, and $s^{Ass}\text{=}A^{ss}/\sum_{r} A^{sr}$.

Substituting $\Delta L^{ss}$for Eq.(A2) in the third term of Eq.(5) and $\text{C}\left(\Delta L^{ss}\right)$ can be further decomposed into two components:

$\begin{matrix} \text{C}\left(\Delta L^{ss}\right)=\frac{1}{2}\left(\hat{\alpha}_{0}^{s}\hat{\beta}_{0}^{s}\Delta L^{ss}S_{1}^{s}+\hat{\alpha}_{1}^{s}\hat{\beta}_{1}^{s}\Delta L^{ss}S_{0}^{s}\right) \\ \begin{matrix} \end{matrix}\begin{matrix} \end{matrix}=\underbrace{\frac{1}{4}\left[\hat{\alpha}_{0}^{s}\hat{\beta}_{0}^{s}L_{1}^{ss}\Delta A^{s}\text{\#}\left(s_{1}^{Ass}+s_{0}^{Ass}\right)L_{0}^{ss}S_{1}^{s}+\hat{\alpha}_{1}^{s}\hat{\beta}_{1}^{s}L_{1}^{ss}\Delta A^{s}\text{\#}\left(s_{1}^{Ass}+s_{0}^{Ass}\right)L_{0}^{ss}S_{0}^{s}\right]}_{effect\begin{matrix} \end{matrix}of\begin{matrix} \end{matrix}\Delta A^{s}} \\ \begin{matrix} \end{matrix}\begin{matrix} \end{matrix}+\underbrace{\frac{1}{4}\left[\hat{\alpha}_{0}^{s}\hat{\beta}_{0}^{s}L_{1}^{ss}\left(A_{1}^{s}+A_{0}^{s}\right)\text{\#}\Delta s^{Ass}L_{0}^{ss}S_{1}^{s}+\hat{\alpha}_{1}^{s}\hat{\beta}_{1}^{s}L_{1}^{ss}\left(A_{1}^{s}+A_{0}^{s}\right)\text{\#}\Delta s^{Ass}L_{0}^{ss}S_{0}^{s}\right]}_{effect\begin{matrix} \end{matrix}of\begin{matrix} \end{matrix}\Delta s^{Ass}} \\ \begin{matrix} \end{matrix}\begin{matrix} \end{matrix}=\text{C}\left(\Delta A^{s}\right)+\text{C}\left(\Delta s^{Ass}\right) \end{matrix}$ (A3)

# Appendix B: Derivation of Equation (7)

For $\text{C}\left(\Delta S^{s}\right)$, as we defined in the previous statement , can be rewritten as:

$S^{s}\text{=}\frac{Y^{ss}+\sum_{r\neq s} A^{sr}X^{r}+\sum_{r\neq s} Y^{sr}+E^{s}}{1'\cdot X_{s}}\text{=}s^{Yss}+r^{A}\cdot\sum_{r\neq s} s^{Asr}+r^{Y}\cdot\sum_{r\neq s} s^{Ysr}+s^{E}$ (B1)

Where $s^{Yss}\text{=}\frac{Y^{ss}}{1'\cdot X_{s}}$, $r^{A}\text{=}\frac{1'\cdot\sum_{r\neq s} A^{sr}X^{r}}{1'\cdot X_{s}}$, $s^{Asr}\text{=}\frac{A^{sr}X^{r}}{1'\cdot\sum_{r\neq s} A^{sr}X^{r}}$, $r^{Y}\text{=}\frac{1'\cdot\sum_{r\neq s} Y^{sr}}{1'\cdot X_{s}}$, $s^{Ysr}\text{=}\frac{Y^{sr}}{1'\cdot\sum_{r\neq s} Y^{sr}}$,$s^{E}\text{=}\frac{E^{s}}{1'\cdot X_{s}}$

We apply Eq.(B1) into the fourth term of Eq.(5), we can have the further decomposition of $\text{C}\left(\Delta S^{s}\right)$ as:

$\begin{matrix} C\left(\Delta S^{s}\right)=\underbrace{\frac{1}{2}\left(\hat{\alpha}_{0}^{s}\hat{\beta}_{0}^{s}L_{0}^{ss}+\hat{\alpha}_{1}^{s}\hat{\beta}_{1}^{s}L_{1}^{ss}\right)\Delta s^{Yss}}_{effect\begin{matrix} \end{matrix}of\begin{matrix} \end{matrix}\Delta s^{Yss}}+\underbrace{\frac{1}{4}\left(\hat{\alpha}_{0}^{s}\hat{\beta}_{0}^{s}L_{0}^{ss}+\hat{\alpha}_{1}^{s}\hat{\beta}_{1}^{s}L_{1}^{ss}\right)\Delta\hat{r}^{A}\left(\sum_{r\neq s} s_{1}^{Asr}+\sum_{r\neq s} s_{0}^{Asr}\right)}_{effect\begin{matrix} \end{matrix}of\begin{matrix} \end{matrix}\Delta r^{A}} \\ +\underbrace{\frac{1}{4}\left(\hat{\alpha}_{0}^{s}\hat{\beta}_{0}^{s}L_{0}^{ss}+\hat{\alpha}_{1}^{s}\hat{\beta}_{1}^{s}L_{1}^{ss}\right)\left(\hat{r}_{1}^{A}+\hat{r}_{0}^{A}\right)\Delta\sum_{r\neq s} s^{Asr}}_{effect\begin{matrix} \end{matrix}of\begin{matrix} \end{matrix}\Delta\sum_{r\neq s} s^{Asr}}+\underbrace{\frac{1}{4}\left(\hat{\alpha}_{0}^{s}\hat{\beta}_{0}^{s}L_{0}^{ss}+\hat{\alpha}_{1}^{s}\hat{\beta}_{1}^{s}L_{1}^{ss}\right)\Delta\hat{r}^{Y}\left(\sum_{r\neq s} s_{1}^{Ysr}+\sum_{r\neq s} s_{0}^{Ysr}\right)}_{effect\begin{matrix} \end{matrix}of\begin{matrix} \end{matrix}\Delta r^{Y}} \\ +\underbrace{\frac{1}{4}\left(\hat{\alpha}_{0}^{s}\hat{\beta}_{0}^{s}L_{0}^{ss}+\hat{\alpha}_{1}^{s}\hat{\beta}_{1}^{s}L_{1}^{ss}\right)\left(\hat{r}_{1}^{Y}+\hat{r}_{0}^{Y}\right)\Delta\sum_{r\neq s} s^{Ysr}}_{effect\begin{matrix} \end{matrix}of\begin{matrix} \end{matrix}\Delta\sum_{r\neq s} s^{Ysr}}+\underbrace{\frac{1}{2}\left(\hat{\alpha}_{0}^{s}\hat{\beta}_{0}^{s}L_{0}^{ss}+\hat{\alpha}_{1}^{s}\hat{\beta}_{1}^{s}L_{1}^{ss}\right)\Delta s^{E}}_{effect\begin{matrix} \end{matrix}of\begin{matrix} \end{matrix}\Delta s^{E}} \\ =C\left(\Delta s^{Yss}\right)+\text{C}\left(\Delta r^{A}\right)+\text{C}\left(\Delta\sum_{r\neq s} s^{Asr}\right)+\text{C}\left(\Delta r^{Y}\right)+\text{C}\left(\Delta\sum_{r\neq s} s^{Ysr}\right)\text{+C}\left(\Delta s^{E}\right) \end{matrix}$ (B2)

Where $\hat{r}^{A}$ is the $n\times n$ diagonal matrix with all diagonal elements equal to $r^{A}$, and $\hat{r}^{Y}$ is similar to $\hat{r}^{A}$.

# Appendix C: Derivation of Equation (12)

For $\text{C}\left[\Delta\left(G^{s}\right)^{'}\right]$, the change of local Ghosh inverse matrix $\Delta\left(G^{s}\right)^{'}$ can be rewritten as the following formula according to its definition:

$\Delta\left(G^{s}\right)^{'}=\left(G_{1}^{ss}\right)^{'}-\left(G_{0}^{ss}\right)^{'}=\left(G_{1}^{ss}\Delta H^{ss}G_{0}^{ss}\right)^{'}=\left(G_{0}^{ss}\right)^{'}\left(\Delta H^{ss}\right)^{'}\left(G_{1}^{ss}\right)^{'}$ (C1)

We substitute $H^{ss}$ for $H^{ss}\text{=}\left(\hat{X}^{s}\right)^{-1}\cdot Z^{ss}\text{=}\left(\hat{X}^{s}\right)^{-1}\cdot Z^{s}\text{\#}r_{z}^{ss}$ (where $Z^{s}\text{=}\sum_{r} Z^{rs}$, $r^{Zss}\text{=}Z^{ss}/\sum_{r} Z^{rs}$) in Eq.(C1) and apply it into the third term of eq.(11), and then we have:

$\begin{matrix} C\left[\Delta\left(G^{s}\right)^{'}\right]\text{=}\frac{1}{2}\left[\hat{\alpha}_{0}^{s}\hat{\beta}_{0}^{s}\Delta\left(G^{s}\right)^{'}D_{1}^{s}+\hat{\alpha}_{1}^{s}\hat{\beta}_{1}^{s}\Delta\left(G^{s}\right)^{'}D_{0}^{s}\right] \\ \text{=}\underbrace{\frac{1}{4}\left\{\begin{matrix} \hat{\alpha}_{0}^{s}\hat{\beta}_{0}^{s}\left(G_{0}^{s}\right)^{'}\left[\Delta\left(\hat{X}^{s}\right)^{-1}Z_{1}^{s}\text{\#}r_{1}^{Zss}+\Delta\left(\hat{X}^{s}\right)^{-1}Z_{0}^{s}\text{\#}r_{0}^{Zss}\right]^{'}\left(G_{1}^{s}\right)^{'}D_{1}^{s} \\ +\hat{\alpha}_{1}^{s}\hat{\beta}_{1}^{s}\left(G_{0}^{s}\right)^{'}\left[\Delta\left(\hat{X}^{s}\right)^{-1}Z_{1}^{s}\text{\#}r_{1}^{Zss}+\Delta\left(\hat{X}^{s}\right)^{-1}Z_{0}^{s}\text{\#}r_{0}^{Zss}\right]^{'}\left(G_{1}^{s}\right)^{'}D_{0}^{s} \end{matrix}\right\}}_{effect\begin{matrix} \end{matrix}of\begin{matrix} \end{matrix}\Delta\left(\hat{X}^{s}\right)^{-1}} \\ \text{+}\underbrace{\frac{1}{4}\left\{\begin{matrix} \hat{\alpha}_{0}^{s}\hat{\beta}_{0}^{s}\left(G_{0}^{s}\right)^{'}\left[\left(\hat{X}_{1}^{s}\right)^{-1}\Delta Z^{ss}\text{\#}r_{0}^{Zss}+\left(\hat{X}_{0}^{s}\right)^{-1}\Delta Z^{ss}\text{\#}r_{1}^{Zss}\right]^{'}\left(G_{1}^{s}\right)^{'}D_{1}^{s} \\ +\hat{\alpha}_{1}^{s}\hat{\beta}_{1}^{s}\left(G_{0}^{s}\right)^{'}\left[\left(\hat{X}_{1}^{s}\right)^{-1}\Delta Z^{ss}\text{\#}r_{0}^{Zss}+\left(\hat{X}_{0}^{s}\right)^{-1}\Delta Z^{ss}\text{\#}r_{1}^{Zss}\right]^{'}\left(G_{1}^{s}\right)^{'}D_{0}^{s} \end{matrix}\right\}}_{effect\begin{matrix} \end{matrix}of\begin{matrix} \end{matrix}\Delta Z^{s}} \\ \text{+}\underbrace{\frac{1}{4}\left\{\begin{matrix} \hat{\alpha}_{0}^{s}\hat{\beta}_{0}^{s}\left(G_{0}^{s}\right)^{'}\left[\left(\hat{X}_{1}^{s}\right)^{-1}Z_{1}^{s}\text{\#}\Delta r^{Zss}+\left(\hat{X}_{0}^{s}\right)^{-1}Z_{0}^{s}\text{\#}\Delta r^{Zss}\right]^{'}\left(G_{1}^{s}\right)^{'}D_{1}^{s} \\ +\hat{\alpha}_{1}^{s}\hat{\beta}_{1}^{s}\left(G_{0}^{s}\right)^{'}\left[\left(\hat{X}_{1}^{s}\right)^{-1}Z_{1}^{s}\text{\#}\Delta r^{Zss}+\left(\hat{X}_{0}^{s}\right)^{-1}Z_{0}^{s}\text{\#}\Delta r^{Zss}\right]^{'}\left(G_{1}^{s}\right)^{'}D_{0}^{s} \end{matrix}\right\}}_{effect\begin{matrix} \end{matrix}of\begin{matrix} \end{matrix}\Delta r^{Zss}} \\ =\text{C}\left[\Delta\left(\hat{X}^{s}\right)^{-1}\right]+\text{C}\left(\Delta Z^{s}\right)+\text{C}\left(\Delta r^{Zss}\right) \end{matrix}$ (C2)

# Appendix D: Derivation of Equation (13)

For $\text{C}\left(\Delta D^{s}\right)$, we rearrange the formula of $D^{s}$, and we have:

$D^{s}\text{=}\frac{\left(\sum_{r\neq s} Z^{rs}\right)^{'}\cdot1+\left(VA^{s}\right)^{'}+\left(IM^{s}\right)^{'}}{1'\cdot X_{s}}=r^{Z}\cdot s^{Zrs}+s^{V}+s^{I}$ (D1)

Where $r^{Z}\text{=}\frac{1'\cdot\left(\sum_{r\neq s} Z^{rs}\right)^{'}}{1'\cdot X^{s}}$，$s^{Zrs}\text{=}\frac{\left(Z^{rs}\right)^{'}\cdot1}{1'\cdot\left(\sum_{r\neq s} Z^{rs}\right)^{'}}$，$s^{V}\text{=}\frac{VA_{t}^{s}}{1'\cdot X_{s}}$，$s^{I}\text{=}\frac{IM_{t}^{s}}{1'\cdot X_{s}}$

Applying Eq.(D1) into the fourth term of Eq.(11), we have:

$\begin{matrix} \text{C}\left(\Delta D^{s}\right)=\underbrace{\frac{1}{4}\left[\hat{\alpha}_{0}^{s}\hat{\beta}_{0}^{s}\left(G_{0}^{s}\right)^{'}+\hat{\alpha}_{1}^{s}\hat{\beta}_{1}^{s}\left(G_{0}^{s}\right)^{'}\right]\Delta\hat{r}^{Z}\left(\sum_{r\neq s} s_{1}^{Zrs}+\sum_{r\neq s} s_{0}^{Zrs}\right)}_{\text{e}ffect\begin{matrix} \end{matrix}of\begin{matrix} \end{matrix}\Delta r^{Z}} \\ \begin{matrix} \end{matrix}\begin{matrix} \end{matrix}+\underbrace{\frac{1}{4}\left[\hat{\alpha}_{0}^{s}\hat{\beta}_{0}^{s}\left(G_{0}^{s}\right)^{'}+\hat{\alpha}_{1}^{s}\hat{\beta}_{1}^{s}\left(G_{0}^{s}\right)^{'}\right]\left(\hat{r}_{1}^{Z}+\hat{r}_{0}^{Z}\right)\Delta\sum_{r\neq s} s^{Zrs}}_{effect\begin{matrix} \end{matrix}of\begin{matrix} \end{matrix}\Delta\sum_{r\neq s} s^{Zrs}} \\ \begin{matrix} \end{matrix}\begin{matrix} \end{matrix}+\underbrace{\frac{1}{2}\left[\hat{\alpha}_{0}^{s}\hat{\beta}_{0}^{s}\left(G_{0}^{s}\right)^{'}+\hat{\alpha}_{1}^{s}\hat{\beta}_{1}^{s}\left(G_{0}^{s}\right)^{'}\right]\Delta s^{V}}_{effect\begin{matrix} \end{matrix}of\begin{matrix} \end{matrix}\Delta s^{V}}+\underbrace{\frac{1}{2}\left[\hat{\alpha}_{0}^{s}\hat{\beta}_{0}^{s}\left(G_{0}^{s}\right)^{'}+\hat{\alpha}_{1}^{s}\hat{\beta}_{1}^{s}\left(G_{0}^{s}\right)^{'}\right]\Delta s^{I}}_{effect\begin{matrix} \end{matrix}of\begin{matrix} \end{matrix}\Delta s^{I}} \\ \begin{matrix} \end{matrix}\begin{matrix} \end{matrix}=\text{C}\left(\Delta r^{Z}\right)+\text{C}\left(\Delta\sum_{r\neq s} s^{Zrs}\right)+\text{C}\left(\Delta s^{V}\right)+\text{C}\left(\Delta s^{I}\right) \end{matrix}$ (D2)
